# Exceptionally Extensive Trevor’s Disease Involving All Four Radial Carpals Presenting With Gross Wrist Deformity

**DOI:** 10.7759/cureus.101327

**Published:** 2026-01-12

**Authors:** Prabodh Kantiwal, Siddhi Chawla, Amir Suhail, Sandeep Kumar Yadav, Meenakshi Rao

**Affiliations:** 1 Orthopedics, All India Institute of Medical Sciences, Jodhpur, Jodhpur, IND; 2 Radiology, All India Institute of Medical Sciences, Jodhpur, Jodhpur, IND; 3 Pathology, All India Institute of Medical Sciences, Jodhpur, Jodhpur, IND

**Keywords:** carpal bones, dysplasia epiphysealis hemimelica, orthopedics, surgery, surgical excision, trevor’s disease, tumor

## Abstract

Trevor’s disease, also known as dysplasia epiphysealis hemimelica (DEH), is an uncommon developmental disorder in which the epiphyseal cartilage grows asymmetrically, typically affecting the lower limbs. Carpal involvement is exceptionally uncommon. We report a four-year-old girl presenting with a progressively enlarging, firm swelling over the volar-radial aspect of the wrist, causing deformity and restricted motion. Imaging revealed multilobulated osteocartilaginous lesions involving the scaphoid, lunate, trapezium, and trapezoid, as well as an additional lesion within the abductor pollicis longus tendon sheath. Histopathology confirmed the diagnosis of DEH. Surgical excision with deformity correction resulted in good functional recovery without recurrence at six months. This rare case of extensive, multilobulated carpal DEH underscores the importance of recognizing its epiphyseal origin to differentiate it from osteochondroma and guide appropriate management.

## Introduction

Dysplasia epiphysealis hemimelica (DEH), also known as Trevor’s disease, is a rare developmental disorder characterized by asymmetric overgrowth of epiphyseal cartilage. First described by Mouchet and Belot in 1926 and later termed “hemimelic epiphyseal dysplasia” by Trevor in 1950, the condition is estimated to occur in approximately 1 in 1,000,000 children [1,2]. It usually involves the lower extremities (80-90% of cases), most frequently the ankle and knee, whereas involvement of the upper extremities (~10-20% of cases) and carpal joints is exceedingly uncommon (<1-2%) [3,4]. DEH is characterized by asymmetric osteocartilaginous overgrowth in the epiphysis, which typically progresses during skeletal growth and ceases at skeletal maturity or puberty [5]. Clinically, DEH may resemble osteochondroma, a far more common lesion that arises from the metaphysis of long bones. However, DEH originates from the epiphysis and usually presents in childhood with localized swelling, deformity, and restricted range of motion. Diagnosis is often delayed or misinterpreted as osteochondroma, enchondroma, or parosteal chondroma [6,7].

Carpal involvement is rare, with fewer than 20 cases reported in the literature to date [8-14]. Most reports describe solitary scaphoid or lunate lesions; synchronous involvement of multiple carpal bones in a pediatric patient is extraordinary [15].

We report a rare case of multilobular DEH affecting the scaphoid, lunate, trapezium, trapezoid, and abductor pollicis longus tendon in a four-year-old girl, managed surgically with an excellent outcome. This case also emphasizes the importance of differentiating DEH from osteochondroma.

## Case presentation

**Figure 1:**
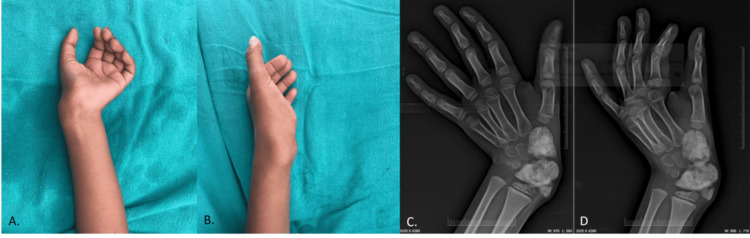
(A, B) Clinical images showing the swelling on the lateral and volar aspects of the left wrist. (C, D) X-ray AP and oblique views demonstrate an expansile sclerotic lesion involving the scaphoid, lunate, trapezium, trapezoid, and the inferolateral aspect of the scaphoid, with the capitate, hamate, and triquetral bones appearing normal.

A four-year-old right-handed girl presented to our outpatient department with a gross, rigid deformity of the left wrist. The wrist was fixed in extension with ulnar deviation, and a firm, bony swelling was noted over the volar-radial aspect of the wrist, extending toward the distal forearm (Figure 1A, 1B). The deformity was associated with limited thumb motion and reduced grip strength. The parents first noticed the swelling at age two, with gradual progression over the following two years. There was no history of trauma or similar lesions in any family member.

On physical examination, the wrist was markedly deformed (Figure 1A, 1B), with approximately 30° of ulnar deviation and extension. The swelling was hard on palpation, multilobulated, non-tender, and immobile relative to the overlying skin and surrounding tendons. The flexor carpi radialis tendon was prominent, and there was visible atrophy of the thenar eminence. Distal neurovascular examination was normal.

**Figure 2:**
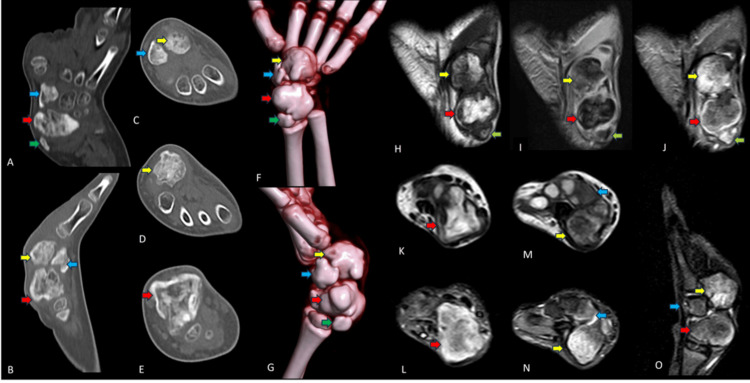
CT images in (A) coronal oblique, (B) sagittal reformatted, (C, D) axial sections at the level of the distal carpal bones, and (E) axial section at the level of the proximal carpal row demonstrate expansile sclerotic lesions in the trapezium (blue arrow), trapezoid (yellow arrow), scaphoid (red arrow), and along the inferolateral margin of the scaphoid (green arrow). Volume-rendered images show similar lesions in (F) AP and (G) lateral views. MRI coronal images depict hyperintense cartilage caps in the trapezoid (yellow arrow), scaphoid (red arrow), and along the abductor pollicis longus tendon (green arrow) in (H) T1-weighted, (I) T2-weighted, and (J) fat-suppressed T2-weighted sequences. Axial images at the level of the proximal carpal row show scaphoid lesions (red arrow) in (K) T1-weighted and (L) fat-saturated T2-weighted sequences. At the level of the distal carpal row, lesions are seen in the trapezium (blue arrow) and trapezoid (yellow arrow) in (M) T1-weighted and (N) fat-saturated T2-weighted axial sections. (O) Sagittal fat-saturated STIR image demonstrates lesions in the trapezium (blue arrow), trapezoid (yellow arrow), and scaphoid (red arrow).

Routine laboratory investigations were within normal limits. Plain radiographs of the left wrist demonstrated multiple expansile, densely sclerotic, multilobulated lesions with irregular margins involving the scaphoid, lunate, trapezium, and trapezoid (Figure 1C, 1D). CT further delineated expansile, sclerotic, “popcorn-like” osseous formations arising from the scaphoid, lunate, trapezium, and trapezoid. MRI demonstrated heterogeneous sessile lesions originating from these carpal bones, along with a smaller lesion adjacent to the scaphoid extending into the abductor pollicis longus tendon sheath, characterized by a hypointense cartilaginous rim. Based on these imaging findings, a benign cartilaginous lesion was suspected, with differential diagnoses including sessile Trevor disease, osteochondroma, and parosteal chondroma (Figure 2A-2O).

**Figure 3:**
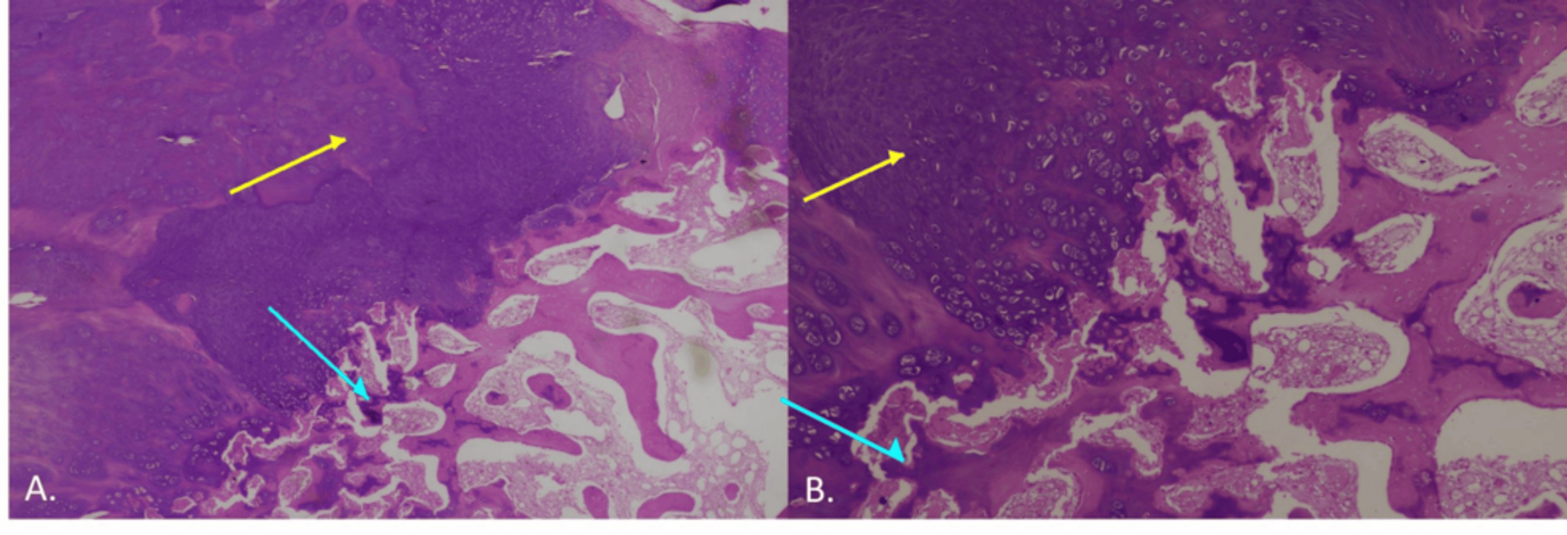
Histopathological examination. (A) Scanner view showing a tumor composed of a cartilaginous cap (yellow arrow) overlying mature bony trabeculae (cyan arrow) (H&E, ×40). (B) Low-power view demonstrating a cartilaginous cap with bland, evenly distributed chondrocytes (yellow arrow) overlying mature trabecular bone (cyan arrow) (H&E, ×100).

Histopathologic evaluation was initially performed on an image-guided percutaneous core needle bone biopsy obtained under fluoroscopic guidance. The biopsy demonstrated a lesion composed of a mature hyaline cartilage cap overlying a bony base of mature trabeculae, without cytologic atypia or abnormal cellular proliferation, consistent with DEH. These findings were subsequently confirmed on histopathological examination of the excised specimen (Figure 3A, 3B).

Debulking and resection of the lesions were performed under general anesthesia through an extensive volar approach. After incising a thick fibrous capsule, multiple lobulated osteocartilaginous masses were identified arising from the volar and radial aspects of the scaphoid, trapezium, and trapezoid. An additional lesion was noted within the abductor pollicis longus tendon sheath. Each lesion had a well-defined cartilage cap.

**Figure 4:**
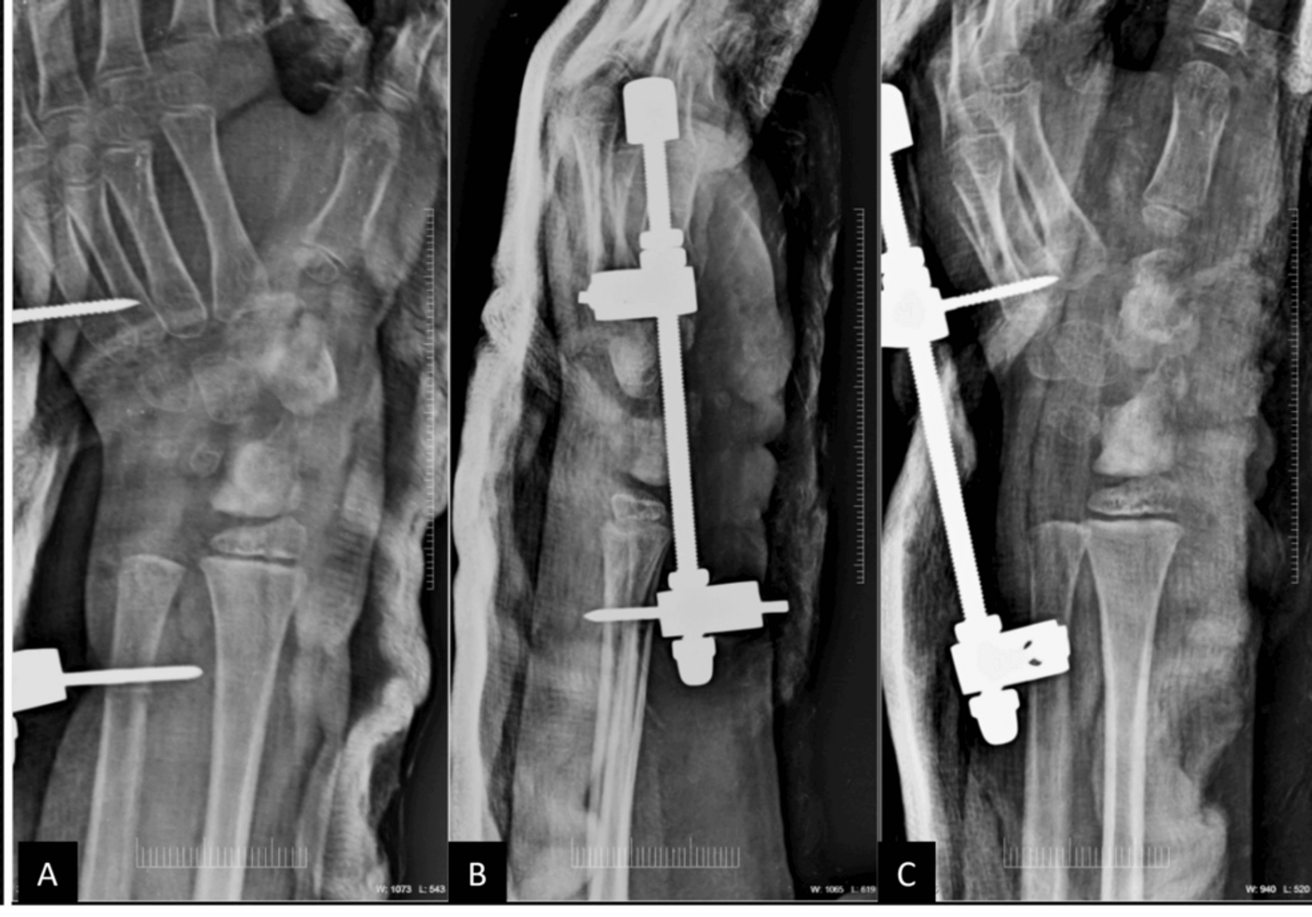
Postoperative X-rays. (A) AP view, (B) lateral view, and (C) oblique view showing reduced size of the carpal bones following tumor debulking, with removal of the lesion in the abductor pollicis longus tendon sheath and placement of the distraction apparatus.

Under fluoroscopic guidance, the lesions were excised with careful preservation of the surrounding carpal bone architecture and adjacent soft tissues. Following tumor debulking, a distraction apparatus was applied (Figure 4A-4C) and maintained for six weeks, after which the patient’s range of motion improved. After removal of the distraction apparatus, a supervised physiotherapy program was initiated, focusing on gradual restoration of wrist and thumb range of motion, gentle strengthening exercises, and prevention of stiffness.

At discharge, the patient was advised to avoid heavy lifting, repetitive wrist strain, and high-impact activities involving the affected hand for at least six weeks. The family was counseled regarding the possibility of lesion regrowth until epiphyseal fusion at puberty and the need for regular clinical and radiological follow-up.

## Discussion

DEH is a rare developmental disorder of the epiphysis that typically presents in early childhood. It results from abnormal proliferation of cartilage arising from the epiphysis, leading to asymmetric growth and formation of intra-articular or periarticular osteocartilaginous masses [1,2]. The lower extremity is most commonly affected, particularly the ankle and knee, whereas involvement of the upper extremity is uncommon, and carpal involvement is exceedingly rare [3,4].

Radiological evaluation is central to the diagnosis of DEH and to differentiating it from other benign cartilaginous lesions. Plain radiographs typically demonstrate asymmetric, irregular epiphyseal overgrowth appearing as a lobulated, expansile osteocartilaginous mass contiguous with the epiphysis. These lesions may appear sclerotic or partially ossified and can result in joint incongruity or deformity, while adjacent carpal bones often remain uninvolved, as observed in the present case (Figure 1).

CT allows superior assessment of the osseous component and is particularly valuable in the wrist because of the complex anatomy of the carpal bones. CT clearly demonstrates the epiphyseal origin of the lesion, defines cortical involvement, and accurately delineates multilobulated lesions affecting multiple carpal bones, features essential for surgical planning (Figure 2).

MRI is the most sensitive modality for evaluating DEH. MRI typically reveals a lobulated epiphyseal lesion with a cartilage cap that is hypointense to isointense on T1-weighted images and hyperintense on T2-weighted and fat-suppressed sequences. MRI also allows accurate assessment of cartilage cap thickness, intra-articular extension, physeal involvement, and associated soft-tissue or tendon sheath extension. In the present case, MRI clearly demonstrated cartilage caps involving multiple carpal bones with extension into the abductor pollicis longus tendon sheath (Figure 2).

Radiologically, DEH has been classified by Azouz et al. based on the distribution of epiphyseal involvement into three types: (1) localized type, involving a single epiphysis; (2) classic type, involving multiple epiphyses within the same limb; and (3) generalized type, involving extensive epiphyseal disease throughout a limb [16]. The localized type is most frequently reported, while the generalized type is extremely rare. Most reported upper-extremity cases fall within the localized or classic categories. Based on this classification, the present case represents the classic type, with synchronous involvement of multiple carpal bones.

The clinical and radiological features of DEH often mimic osteochondroma, the most common benign bone tumor. However, DEH arises from the epiphysis and demonstrates an asymmetric, hemimelic growth pattern, whereas osteochondromas typically arise from the metaphysis and rarely involve the carpal bones [6]. Histologically, both lesions appear similar, showing a cartilaginous cap overlying trabecular bone, making radiographic localization and clinical context crucial for accurate diagnosis [7].

Carpal involvement in DEH was first reported in 1949 [8]. Since then, only a limited number of cases involving the carpal bones have been documented, mostly as isolated case reports with heterogeneous presentations and variable follow-up durations [9,10]. Subsequent reports described involvement of the distal ulna, scaphoid, and lunate in children, with surgical excision resulting in restoration of wrist motion [11,12]. More recent publications have confirmed favorable outcomes following excision, and a small case series reported good functional results with both surgical and conservative management in selected patients [4,13,14]. A summary of all previously reported cases of carpal DEH, including age, bones involved, management, and outcomes, is provided in Table 1, highlighting the extreme rarity of this condition and the predominance of isolated or limited carpal involvement.

**Table 1:** Reported cases of DEH involving carpal bones DEH, dysplasia epiphysealis hemimelica

Year	Author	Age/sex	Bone(s) involved	Management	Outcome
1949	Geschickter [8]	1 child	Scaphoid	Not detailed	Not reported
1964	Saxton et al. [9]	1 child	Scaphoid, trapezium	Not detailed	Not reported
1973	Wynne-Davies [10]	1 case	Carpus (unspecified)	Not detailed	Not reported
1978	Buckwalter et al. [11]	3 years, M	Distal ulna, lunate	Excision	Full motion, no recurrence
1983	Lamesch [12]	11 years, F	Scaphoid, lunate	Excision	Good motion, no recurrence
1999	Vanhoenacker et al. [3]	Child	Scaphoid	Not detailed	No follow-up
2005	Beer et al. [4]	3 children	Carpal bones (varied)	Excision/observation	Good function
2009	Vogel et al. [13]	7 years, M	Scaphoid	Excision	Full recovery
2019	Ali et al. [14]	16 years, M	Lunate	Excision + curettage	Pain-free, good outcome
2025	Our case	4 years, F	Scaphoid, lunate, trapezium, trapezoid, abductor pollicis longus tendon	Excision	Pain-free, good outcome

The present case expands the existing literature by documenting extensive synchronous multilobulated involvement of the scaphoid, lunate, trapezium, and trapezoid, with extension into the abductor pollicis longus tendon sheath, in a four-year-old child. Such extensive multi-carpal involvement at this young age has not been previously reported. This case underscores how DEH can closely mimic multiple osteochondromas when several carpal bones are involved simultaneously.

The differential diagnosis in such cases primarily includes osteochondroma, parosteal chondroma, and enchondroma. Differentiating DEH from osteochondroma is particularly challenging due to overlapping clinical and histological features. The key distinction lies in the anatomical origin: epiphyseal in DEH and metaphyseal in osteochondroma. Although rare, carpal osteochondromas have been reported involving the scaphoid, trapezium, capitate, and trapezoid, and they may lead to complications such as tendon rupture, painful snapping, arthritis, and nerve compression [6,7]. In contrast, carpal DEH typically presents earlier in childhood and has no reported malignant transformation. The distinguishing clinical, radiological, and pathological features of carpal DEH and carpal osteochondroma are summarized in Table 2, facilitating accurate diagnosis in ambiguous cases.

**Table 2:** Comparison of carpal osteochondroma and carpal DEH DEH, dysplasia epiphysealis hemimelica

Feature	Carpal osteochondroma	Carpal DEH
Origin	Metaphysis of long bones, rarely carpal	Epiphysis of carpal or long bones
Age at presentation	Adolescents/young adults, rarely <10 years	Children (2-14 years), usually <10 years
Sex predilection	Equal or slight male predominance	Male predominance (~3:1)
Lesion number	Solitary; multiple in hereditary cases	Solitary or multiple within one epiphysis
Carpal involvement	Very rare; scaphoid, trapezium, capitate, trapezoid	Very rare; scaphoid, lunate most common
Radiology	Metaphyseal projection, continuous with cortex and medulla	Epiphyseal irregular mass, not continuous with metaphysis
Histology	Cartilage cap with trabecular bone	Similar; epiphyseal location key to diagnosis
Complications	Deformity, tendon rupture, arthritis, rare malignant change	Deformity, growth disturbance, joint dysfunction; malignant change not reported

Management of DEH is guided by symptom severity. Asymptomatic lesions may be observed, particularly in younger children, whereas symptomatic lesions causing pain, deformity, or functional limitation require surgical excision. The literature consistently reports good functional recovery and low recurrence rates following complete excision [11-14]. In the present case, the patient demonstrated significant improvement in wrist motion and function following surgical excision and distraction, with no evidence of recurrence at six-month follow-up.

Overall, this case emphasizes the importance of considering DEH in the differential diagnosis of epiphyseal carpal lesions in children. Early recognition helps prevent misdiagnosis as osteochondroma and allows timely surgical management in symptomatic cases to restore function and prevent long-term disability.

## Conclusions

DEH is a rare developmental disorder of the epiphysis, with carpal involvement being exceptionally uncommon. This case illustrates a unique presentation of multiple synchronous carpal lesions in a four-year-old child, successfully managed with surgical excision, and adds to the limited literature on carpal DEH. Awareness of DEH is essential for distinguishing it from other common bone tumors, guiding appropriate management, and providing an accurate prognosis. Accurate diagnosis relies on the integration of clinical, radiological, and pathological findings.
